# Supplementary material for: High Energy Storage Density in Nanocomposites of P(VDF-TrFE-CFE) Terpolymer and BaZr0.2Ti0.8O3 Nanoparticles
Source: Materials (Basel). 2022 Apr 27;15(9):3151. doi: 10.3390/ma15093151 (PMC9105365; doi:10.3390/ma15093151)
Supplement: Supplementary file 1 [file materials-15-03151-s001.zip › materials-1670370-supplementary.pdf]

Supporting Information

High Energy Storage Density in Nanocomposites of P(VDF-TrFE-CFE) Terpolymer and $BaZr_{0.2}Ti_{0.8}O_3$ Nanoparticles

Yusra Hambal [1], Vladimir V. Shvartsman [1,*], Ivo Michiels [1], Qiming Zhang [2], and Doru C. Lupascu [1]

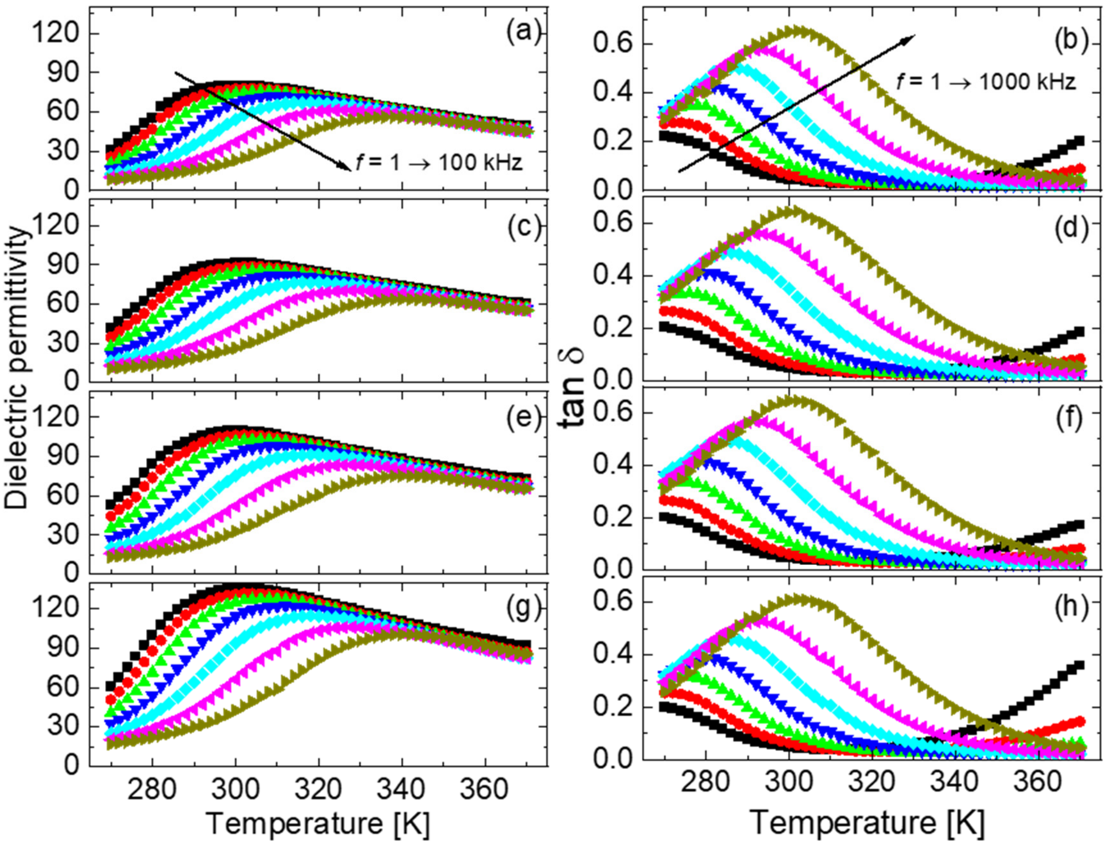

Figure S1. Temperature dependences of dielectric permittivity and dielectric loss tangent of P(VDF-TrFE-CFE) 64.8/35.2/7.8 with varying BZT nanoparticles content, (a, b) 0 vol.%, (c., d) 1.25 vol.%, (e, f) 2.5 vol.% and (g, h) 5 vol.%.

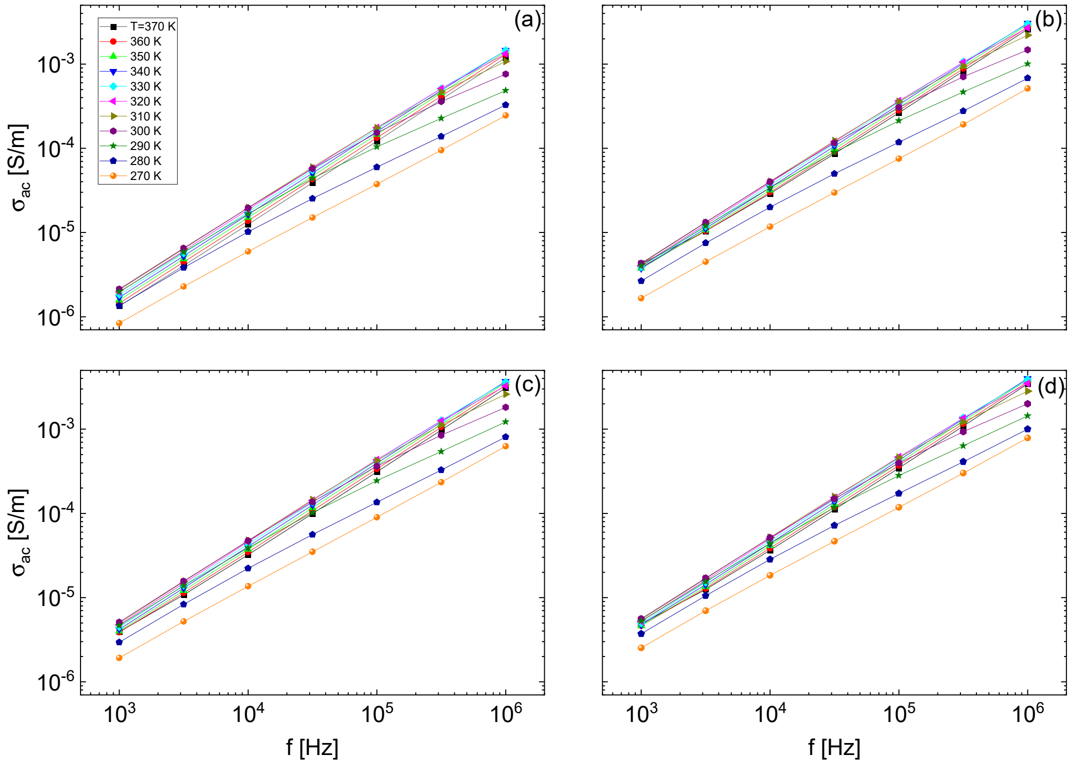

Figure S2. Frequency dependences of ac electric conductivity of P(VDF-TrFE-CFE) 68/32/8.5 composites with 0 vol.% (a), 1.25 vol.% (b), 2.5 vol.% (c), and 5 vol.% (d) of BZT nanoparticles measured at different temperatures.

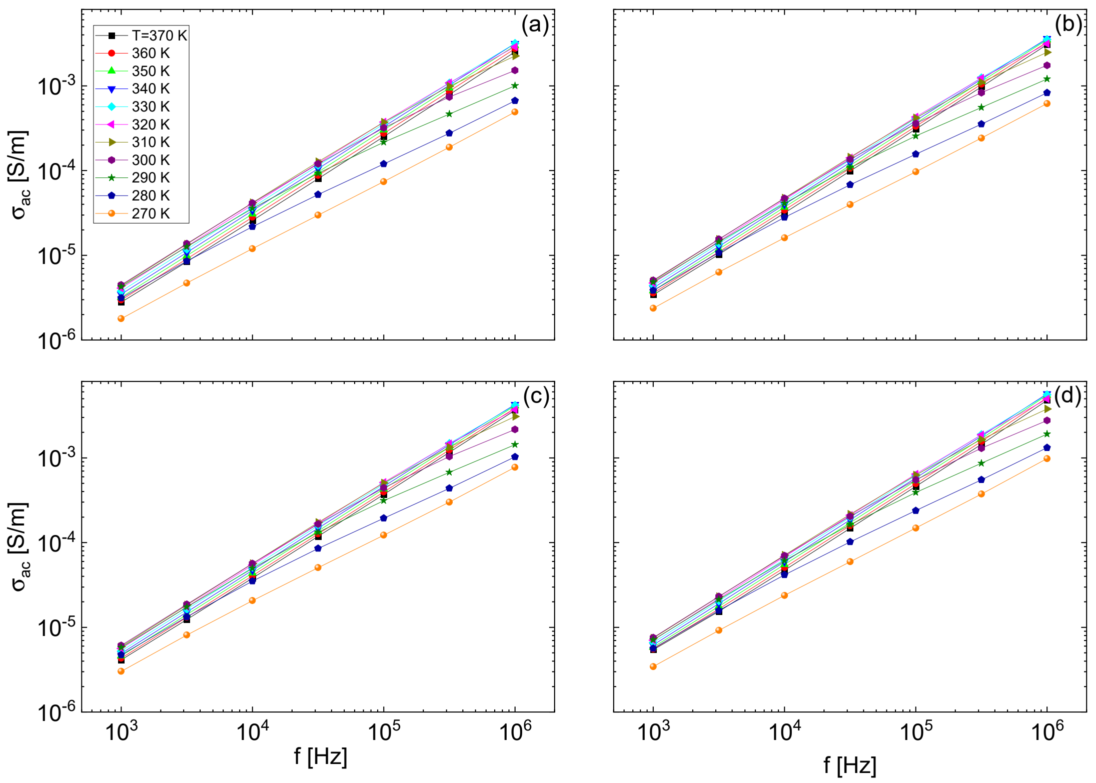

Figure S3. Frequency dependences of ac electric conductivity of P(VDF-TrFE-CFE) 64.8/35.2/7.8 composites with 0 vol.% (a), 1.25 vol.% (b), 2.5 vol.% (c), and 5 vol.% (d) of BZT nanoparticles measured at different temperatures.

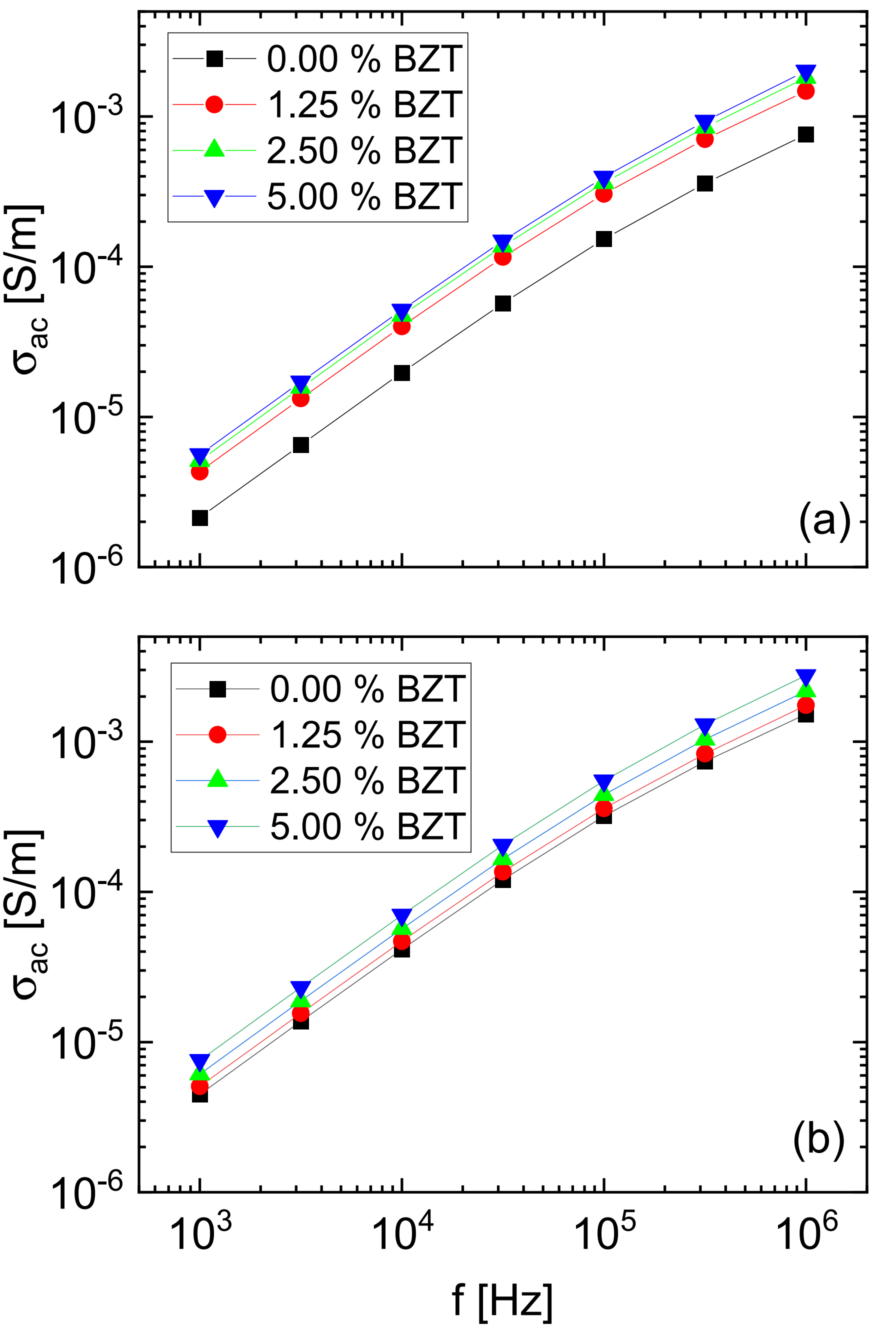

Figure S4. Frequency dependences of ac electric conductivity of P(VDF-TrFE-CFE) 68/32/8.5 (a) and P(VDF-TrFE-CFE) 64.8/35.2/7.8 (b) with varying BZT nanoparticles content measured at 300 K.
